# Fabrication of acoustically and physically validated artificial stones to natural kidney stones under shock waves and laser lithotripsy

**DOI:** 10.1007/s00240-024-01613-z

**Published:** 2024-08-12

**Authors:** Hyeji Park, Sang Won So, Christine Joy G. Castillo, Majed M. Alharthi, Mohammad Mesadef A. Zogan, Sung Yong Cho

**Affiliations:** 1 Department of Urology, Seoul National University Hospital, Seoul, Korea; 2 Department of Urology, Jose R. Reyes Memorial Medical Center, Manila, Philippines; 3 Department of Urology, King Fahd General Hospital, Ministry of Health, Jeddah, Saudi Arabia; 4 Department of Urology, Prince Mohammed bin Nasser Hospital, Jazan, Saudi Arabia; 5 Department of Urology, Seoul National University Hospital, Seoul National University College of Medicine, 101, Daehak-ro, Jongno-gu, Seoul, 03080 Republic of Korea; 6 Innovative Medical Technology Research Institute, Seoul National University Hospital, Seoul, Korea

**Keywords:** Kidney calculi, Lithotripsy, Calcium oxalate, Uric acid, Laser

## Abstract

To present an efficient method for fabricating artificial kidney stones with acoustic and physical properties to assess their fragmentation efficiency under shock waves and laser lithotripsy for very hard stones. The mixture ratio of super-hard plaster and water was adjusted to produce artificial kidney stones for comparison with > 95% human genuine calcium oxalate monohydrate (COM) and uric acid (UA) stones. Acoustic and physical properties, such as wave speed, stone hardness, density, compressive strength, and stone-free rates under shock-wave and laser lithotripsy, were assessed. The longitudinal wave speed of artificial stones prepared at a plaster-to-water ratio of 15:3 closely matched that of COM stones. Similarly, the transverse wave speed of artificial stones prepared at a plaster-to-water ratio of 15:3 to 15:5 aligned with that of COM stones. Stone fragmentation using shock-wave of artificial stones with mixed ratios ranging from 15:3 to 15:5 resembled that of COM stones. The Vickers hardness was similar to that of artificial stones produced with a mixing ratio of 15:3, similar to that of COM stones, while that of artificial stones produced with a mixing ratio of 15:5 was similar to that of UA stones. Density-wise, artificial stones with mixing ratios of 15:4 and 15:5 resembled COM stones. Compressive strength test results did not confirm the similarity between natural and artificial stones. The stone fragmentation using laser showed that stones produced with higher moisture content at a mixing ratio of 15:6 were similar to COM stones. This novel method for fabricating artificial kidney stones could be used to provide reliable materials for lithotripsy research.

## Introduction

The main methods for treating urolithiasis, which is increasingly prevalent worldwide, are shockwave lithotripsy and laser lithotripsy. Extracorporeal shockwave lithotripsy is a non-invasive clinical therapy for treating renal and urinary stones. It operates by focusing shockwaves onto a single point-to-stone [1]. During laser lithotripsy, a laser fiber is positioned endoscopically in direct contact with a kidney stone, enabling the delivery of laser pulses to break down stones within the body and facilitate their removal [2]. Stone fragmentation efficiency during lithotripsy varies depending on some parameters such as laser energy and frequency [3], patient characteristics including anatomical features and overall health status, surgeon expertise [4], stone properties such as size and hardness [5], and shockwave intensity and protocol [6]. Artificial stones are commonly used during the training process for energy parameter setting and endoscopic insertion for urinary stone fragmentation, as well as in evaluating the efficacy of newly developed lithotripters [7]. However, the effectiveness of stone fragmentation training may diminish if artificial stones significantly differ in hardness from real stones, ultimately rendering it difficult to accurately assess the appropriate effectiveness of lithotripters, as these artificial stones fail to reflect the acoustic and physical properties of real stones.

Lithotriptor efficacy assessment is crucial for evaluating the fragmentation ability. It should focus on treating hard stones because hard calculi significantly influence therapeutic outcomes related to the occurrence of residual stones [8]. For less dense stones, the standardized energy setting is important for sufficient lithotripsy for each lithotriptor. Surgeons can determine the setting consistently according to the stone density, and it does not impact the presence of residual fragments. Pure calcium oxalate monohydrate (COM) stones exhibit the highest hardness level among all types of stones [9]. However, the density and distribution of Hounsfield units on CT scans vary significantly depending on the composition of the stone. Therefore, there may need to be more than just predicting stone density based on stone composition.

The acoustic and physical characteristics of stones play a crucial role in determining their interaction during lithotripsy, directly influencing a procedure’s efficacy [10]. Nonetheless, our understanding of artificial stones remains incomplete. Therefore, standardized methods for manufacturing artificial stones mimicking hard human stones that can be used in both shockwave and laser lithotripsy procedures are required. In this study, artificial stones suitable for both in vitro and in vivo applications were fabricated by adjusting super-hard plaster powder composition ratios. The acoustic and physical properties of these artificial stones were thoroughly characterized and compared with those of real stones.

## Materials & methods

### Human stone acquisition

Stones composed entirely or predominantly (at least 95%) of COM and 100% of uric acid stones were obtained from patients who underwent flexible ureteroscopic surgery or percutaneous nephrolithotomy. These stones were utilized to compare acoustic and physical properties against those of artificial stones. This study was approved by our Institutional Review Board (IRB) (IRB approval No. 1901-104-1005).

### Artificial stone preparation

Super-hard plaster powder (Premium Rock, Youngnam, Korea) was mixed with distilled water at ratios of 15:3, 15:4, 15:5, and 15:6 to form a paste. The mixture was degassed twice using a degassing machine to eliminate any bubbles completely. The paste was spread over silicone molds using a spatula and filled at 5 millimeters. All samples were left to dry at room temperature for over 12 h and subsequently removed from the molds.

### Acoustic properties

Velocities of longitudinal and transverse waves were determined to measure the acoustic properties of artificial stones. The spacing between echoes was calculated using the ultrasound pulse-echo method on a 5 mm thick sample. A pair of ultrasonic transducers were affixed to each side of the stone, with one functioning as a transmitter and the other as a receiver. Longitudinal waves were measured with a 10 MHz probe, while transverse waves were measured with a 5 MHz probe. Both were monitored with a digital oscilloscope. Acoustic properties were assessed through eight repetitions of measurement.

### Physical properties

Vickers hardness value was determined by applying a load of 0.20000 kgf and calculating the length of the diagonal and the surface area. Stones were securely mounted and polished to prevent fracture. They were then firmly fixed in an acrylic mold to ensure stability before measurement. Density measurement was conducted by placing stones on the upper part of a density meter to measure their weight and submerging them at a specific point in water to measure their volume. The density was then calculated by dividing the mass by the volume. The compressive strength was measured using a manual, stand-type push-pull gauge equipped with a sharp tip. Physical properties were evaluated through measurements repeated 4 to 10 times.

### Stone fragmentation

Extracorporeal shock wave lithotripsy was performed using an embedded ultrasonic electroconductive Sonolith i-move^®^ shockwave lithotripter (EDAP, Lyon, France). The frequency of lithotripsy was 1 Hz. With an intensity of 75%, the total energy was 254 mJ/mm² over 20 min. Following lithotripsy, the remaining fragments were filtered through a 2 mm sieve, dried, and weighed to measure the stone-free rate.

Laser lithotripsy was performed by tilting individually customized zigs to adjust artificial or human stones to converge at a point of 1–2 mm from the stones. The procedure was conducted at a frequency of 20 pulses per second (Hz), with an energy of 1 J and a duration of 15 min. Similar to extracorporeal shock wave lithotripsy, stones remaining after fragmentation were filtered through a 2 mm sieve, dried, and weighed to measure the stone-free rate.

## Results

**Fig. 1:**
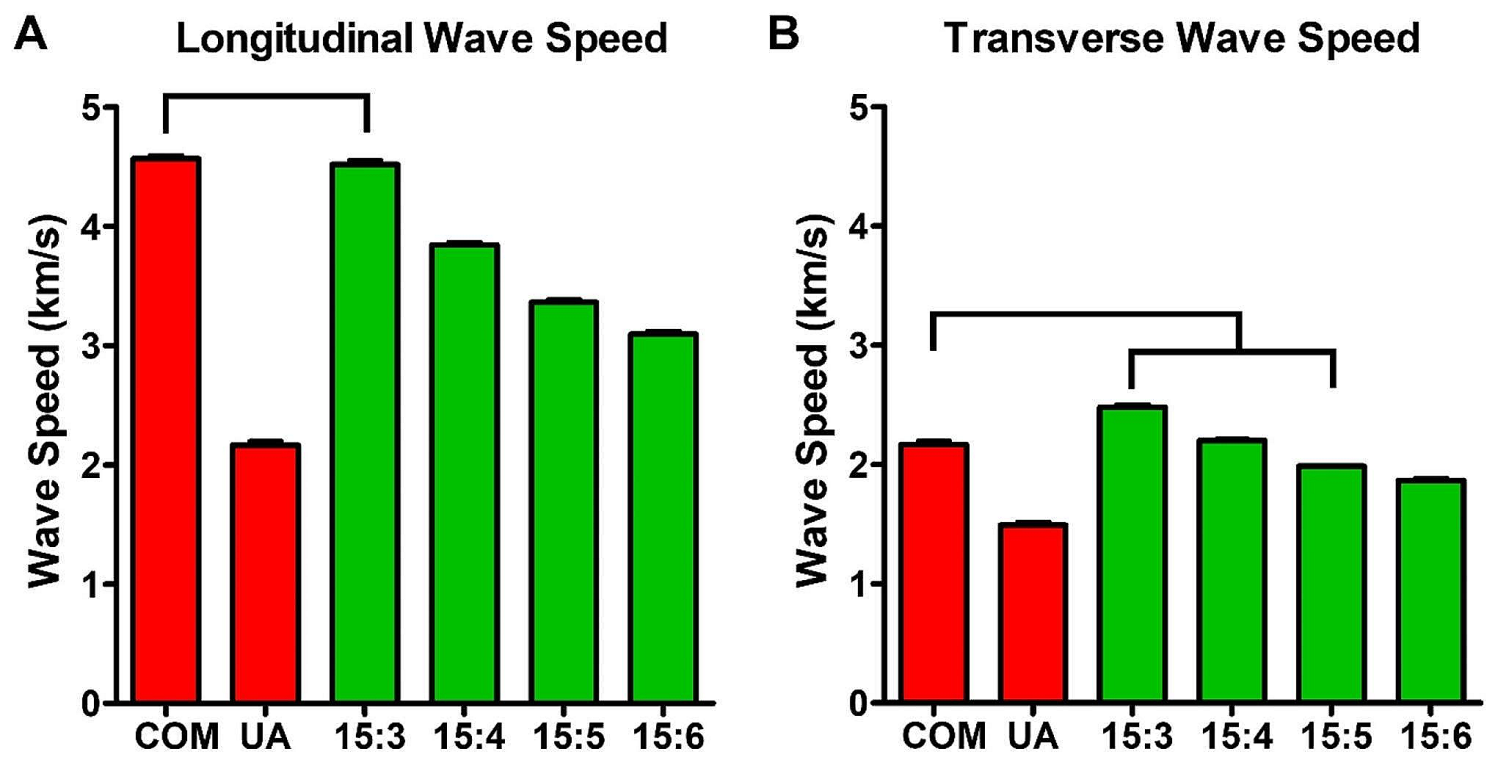
The longitudinal and transverse wave speed of artificial and natural kidney stones

**Fig. 2:**
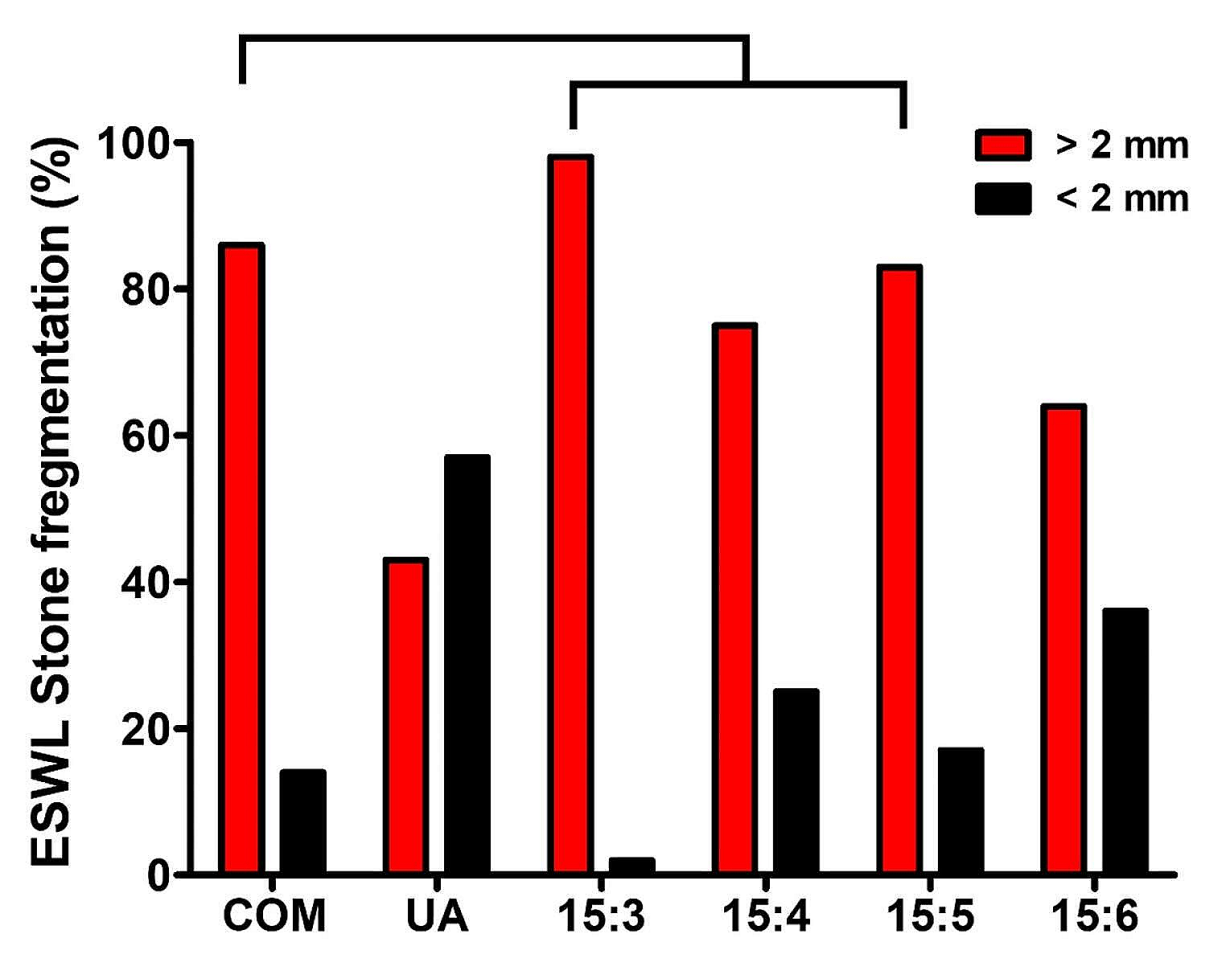
Stone fragmentation efficiency of natural kidney stones and artificial stones by ESWL lithotripsy

The bulk modulus, Young’s modulus, shear modulus, Poisson’s ratio, and longitudinal and transverse wave speeds of artificial stones mixed at ratios ranging from 15:3 to 15:6 of plaster and water are summarized in Table 1. Data measured from actual COM and UA stones were included for comparison purposes. These data for natural kidney stones were obtained from a previous investigation [11]. As shown in Fig. 1a and b, the longitudinal wave speed of artificial stones prepared at a plaster-to-water ratio of 15:3 closely matched that of COM stones. Similarly, the transverse wave speed approximately of artificial stones prepared at a plaster-to-water ratio of 15:3 to 15:5 aligned with that of COM stones. The ESWL stone fragmentation rate of artificial stone mixed with plaster-to-water ratios ranging from 15:3 to 15:5 closely matched that of COM stones, as depicted in Fig. 2.

**Table 1:** Bulk modulus, Young’s modulus, shear modulus, Poisson’s ratio, longitudinal and transverse wave speeds of artificial stones with different ratios, and natural kidney stones

Stone material	Bulk Modulus (Gpa)	Young’s Modulus (Gpa)	Shear Modulus (Gpa)	Poisson’s ratio	Longitudinal wave speed (km/s)	Transverse wave wpeed (km/s)
**COM†**	24.269	24.510	9.200	0.330	4.535 ± 0.058	2.132 ± 0.025
**UA†**	14.200	9.200	3.300	0.390	3.471 ± 0.062	1.464 ± 0.012
**15:3**	25.806	27.282	13.022	0.139	4.519 ± 0.106	2.482 ± 0.076
**15:4**	15.104	17.708	8.775	0.116	3.845 ± 0.077	2.200 ± 0.049
**15:5**	10.440	13.342	6.838	0.098	3.362 ± 0.079	1.988 ± 0.016
**15:6**	7.624	10.308	5.407	0.086	3.094 ± 0.092	1.868 ± 0.056

† Natural kidney stone date from reference [J Urol 2000 Aug;164(2):537 − 44]

**Fig. 3:**
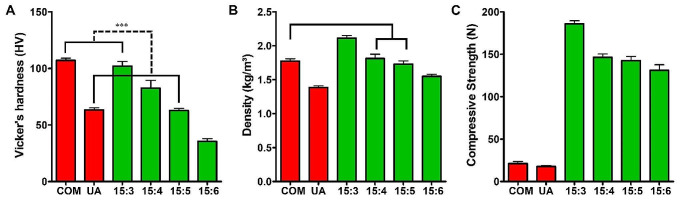
Hardness, density, and compressive strength of natural kidney stones and artificial stones

**Fig. 4:**
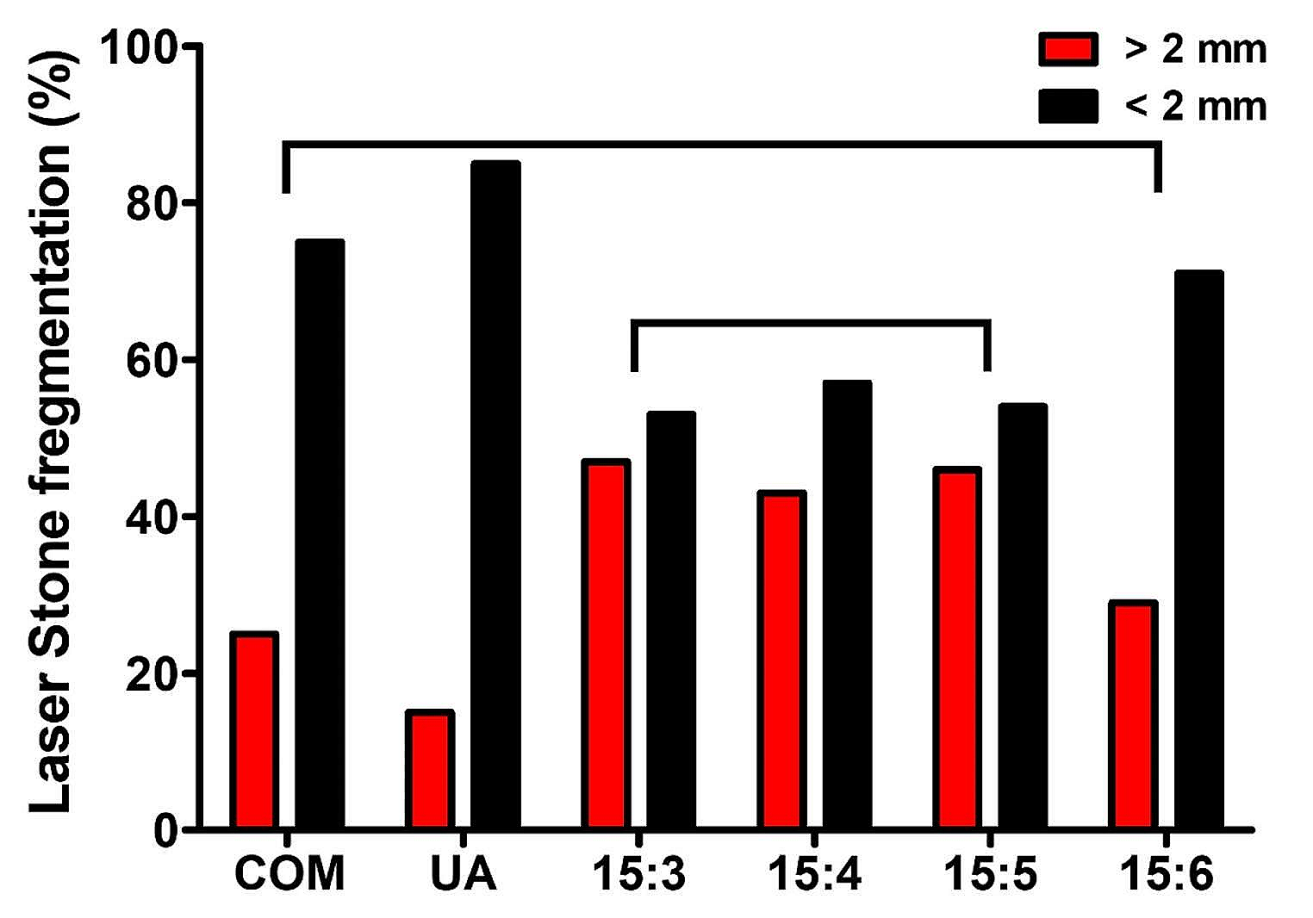
Stone fragmentation efficiency of natural kidney stones and artificial stones by laser lithotripsy

Vickers hardness values of artificial stones produced with a mixing ratio of 15:3 were similar to those of COM stones. These values of artificial stones produced with a mixing ratio of 15:5 were similar to those of UA stones. Density-wise, artificial stones produced with mixing ratios of 15:4 and 15:5 were similar to COM stones. Compressive strength test results did not confirm a similarity between natural and artificial kidney stones (Fig. 3a and c). Results from stone fragmentation using laser showed that stones produced at a mixing ratio of 15:6 with a higher moisture content were similar to COM stones wh. Stones produced with mixing ratios of 15:3, 15:4, and 15:5 exhibited similar fragmentation rates (Fig. 4).

## Discussion

This is the first study that concurrently investigates shockwave and laser lithotripsy on human and artificial stones. Before using a super-hard plaster in this study, artificial stones made of materials such as the plaster of Paris and Begostone were already used in lithotripsy research [12, 13]. However, their acoustic and physical properties have not been fully characterized yet. The super-hard plaster used in this study is easily obtainable. It is the strongest dental plaster. It has the advantage of being stable, with a low expansion rate and a smooth surface due to its refined manufacturing process, which can minimize the formation of bubbles.

While some lithotriptors have been introduced to the market, there have been instances where hard stones remain unfragmented. When the laser is set at the same energy and frequency and irradiated for the same duration, hard stones produced at a mixing ratio of 15:3 with a lower moisture content are hardly fragmented, while soft stones formed at a mixing ratio of 15:12 with a higher moisture content are easily fragmented [14]. Recognizing the necessity of representing hard stones, we aimed to create and validate artificial stones capable of mimicking hard stones, which could be utilized alongside ESWL and laser lithotripsy. In particular, since stones tend to exhibit greater hardness at their core than at the surface, evaluating the fragmentation efficacy of lithotriptors on hard components is crucial for their assessment. Stones form when minerals precipitate and become saturated in the urinary tract, resulting in the formation of microscopic particles that nucleate and aggregate from the center, leading to growth [6]. Therefore, this study focused on obtaining pure COM stones, either 100% or 95%, from human subjects, aiming to compare and analyze them. The fragmentation capacity of less dense stones must be studied. Still, suppose one can effectively break harder stones, it may be more crucial to devise a protocol that can reduce the energy requirement, thereby fragmenting less dense stones to a consistent level. Such settings pose no clinical concerns as long as the lithotriptor provides a stable energy output.

Previous studies have examined the feasibility of artificial stones made from Begostone in ESWL and laser treatments separately. Using BEGO stones, the efficacy and safety of high-frequency shock wave lithotripsy have been measured in a porcine model [15] and the lithotripsy efficacy of thulium Fiber Laser has been evaluated by assessing variations in laser settings using a kidney phantom [16]. This study demonstrates the possibility of creating artificial stones for testing and validating both ESWL and laser treatments simultaneously using only a mixture of plaster and water. Depending exclusively on compressive strength measurements, using a push-pull gauge to evaluate the feasibility of laser fragmentation may not provide accurate assessments of the density or hardness of stones. Therefore, utilizing Vickers hardness tests or densitometry methods is a more suitable methodology [11]. A noteworthy observation was the correlation between results of transverse and longitudinal wave speed analysis for assessing the feasibility of ESWL and outcomes of laser fragmentation results. Therefore, to evaluate lithotriptor performance, two protocols could be employed: one involving the production of very hard stones with a low moisture content ratio of 15:3 and the other aimed at creating softer stones by increasing the moisture content to a ratio of 15:6. Testing the lithotriptor with these two protocols would be appropriate.

While not presented in this study’s findings, the inconsistency observed in the measured stone density at 10 mm and 15 mm was a crucial aspect in the fabrication of artificial stones. This inconsistency was attributed to irregular air bubble formation during preparation, which could disrupt stones’ homogeneity. Larger sizes may lead to inconsistent results in stone characteristic measurements in the fabrication of artificial stones. To minimize the presence of bubbles, artificial stones should be manufactured to a thickness of approximately 5 mm. A degassing chamber is imperative.

### Limitations

The primary limitation of this study was the limited number of stone fragmentation experiments and physical characteristics assessment. However, excluding 10 mm and 15 mm stones from the analysis yielded consistent results, highlighting the importance of establishing a protocol for creating 5 mm stones and performing degassing. The second limitation of this paper was its inability to delineate the manufacturing methods of artificial stones for each constituent in detail.

## Conclusions

To evaluate a lithotriptor’s performance by producing artificial stones, it was concluded that to create very hard stones, a mixture ratio of 15:3 of super-hard plaster to water should be used. For softer stones, the ratio should be 15:6. Testing a lithotriptor using these two protocols would be appropriate.

## Data Availability

No datasets were generated or analysed during the current study.

## Abbreviations

COM: Calcium Oxalate Monohydrate
UA: Uric Acid
